# Draft Genome Sequence of the Carrageenan-Degrading Bacterium *Cellulophaga* sp. Strain KL-A, Isolated from Decaying Marine Algae

**DOI:** 10.1128/genomeA.00145-14

**Published:** 2014-03-06

**Authors:** Dapeng Shan, Jianxi Ying, Xu Li, Zheng Gao, Guangshan Wei, Zongze Shao

**Affiliations:** a Key Laboratory of Marine Biogenetic Resources, Third Institute of Oceanography, State Oceanic Administration, Xiamen, People’s Republic of China; b State Key Laboratory of Crop Biology, College of Life Sciences, Shandong Agricultural University, Taian, Shandong, People’s Republic of China

## Abstract

*Cellulophaga* sp. strain KL-A, isolated from decaying marine algae, is able to degrade iota-carrageenan. Here, we report the draft genome sequence of *Cellulophaga* sp. strain KL-A.

## GENOME ANNOUNCEMENT

*Cellulophaga* sp. strain KL-A is a Gram-positive, aerobic, nonmotile, mesophilic strain isolated from decaying marine algae. During screening, it was found that this strain is capable of degrading iota-carrageenan. Carrageenans are composed of alternating 3-linked β-D-galactopyranose and 4-linked α-D-galactopyranose residues, forming disaccharide repeating units (1). Iota-carrageenan can be specifically hydrolyzed by iota-carrageenase (2). *Cellulophaga* sp. strain KL-A grows well in marine 2216E medium with iota-carrageenan, and it can form a clear zone around the colony by flooding the solid medium with 10% cetylpyridinium chloride.

Here, we present the genome sequence of *Cellulophaga* sp. strain KL-A, which was obtained using Solexa paired-end sequencing technology (3) by Shanghai Majorbio Bio-pharm Technology Co., Ltd. (Shanghai, China). A library with a fragment length of 300 bp was constructed, and a total of 6,681,049 paired-end reads were generated, which provided a 278-fold depth of coverage, with an Illumina/Solexa Genome Analyzer IIx (4) (Illumina, San Diego, CA). The gaps among the scaffolds (5) were closed by custom primer walks or by PCR amplification followed by sequencing. The genome sequence of *Cellulophaga* sp. KL-A comprises 3,888,471 bp, with an average G+C content of 31.94%, consisting of 110 contigs (N_50_, 140,277 bp). Automatic gene annotation was carried out by the NCBI Prokaryotic Genomes Automatic Annotation Pipeline (PGAAP) (http://www.ncbi.nlm.nih.gov/genomes/static/Pipeline.html) and was followed by manual editing. The genome sequence contains 3,503 candidate protein-coding genes, giving a coding intensity of 90.6%, and the average size of each gene is 1,005 bp. A total of 1,415 proteins were assigned to Clusters of Orthologous Groups (COG) families (6). In addition, 32 tRNA genes for 20 amino acids and one 16S-23S-5S rRNA operon were identified in the genome.

The genes possibly responsible for carrageen degradation were analyzed in the genome sequence of *Cellulophaga* sp. KL-A. Two open reading frames (ORFs), ORF01064 and ORF00666, encode the putative carrageenases. Furthermore, there are 3 ORFs (ORF00682, ORF02006, and ORF02026) encoding putative beta-agarase, according to the annotation. The genome information and annotation reported in the present study are valuable for future research to investigate carrageenan degradation in marine environments.

### Nucleotide sequence accession numbers.

This whole-genome shotgun project has been deposited at DDBJ/EMBL/GenBank under the accession no. ARZX00000000. The version described in this paper is version ARZX01000000.
